# Simulations of increased glomerular capillary wall strain in the 5/6‐nephrectomized rat

**DOI:** 10.1111/micc.12721

**Published:** 2021-07-11

**Authors:** Owen Richfield, Ricardo Cortez, L. Gabriel Navar

**Affiliations:** ^1^ Bioinnovation PhD Program Tulane University New Orleans LA USA; ^2^ Department of Physiology Tulane School of Medicine New Orleans LA USA; ^3^ Department of Mathematics Tulane University New Orleans LA USA

**Keywords:** glomerulus, mathematical modeling, mechanical stretch, renal hemodynamics, wall strain

## Abstract

**Objective:**

Chronic glomerular hypertension is associated with glomerular injury and sclerosis; however, the mechanism by which increases in pressure damage glomerular podocytes remains unclear. We tested the hypothesis that increases in glomerular pressure may deleteriously affect podocyte structural integrity by increasing the strain of the glomerular capillary walls, and that glomerular capillary wall strain may play a significant role in the perpetuation of glomerular injury in disease states that are associated with glomerular hypertension.

**Methods:**

We developed an anatomically accurate mathematical model of a compliant, filtering rat glomerulus to quantify the strain of the glomerular capillary walls in a remnant glomerulus of the 5/6‐nephrectomized rat model of chronic kidney disease. In terms of estimating the mechanical stresses and strains in the glomerular capillaries, this mathematical model is a substantial improvement over previous models which do not consider pressure‐induced alterations in glomerular capillary diameters in distributing plasma and erythrocytes throughout the network.

**Results:**

Using previously reported data from experiments measuring the change of glomerular volume as a function of perfusion pressure, we estimated the Young's modulus of the glomerular capillary walls in both control and 5/6‐nephrectomized conditions. We found that in 5/6‐nephrectomized conditions, the Young's modulus increased to 8.6 MPa from 7.8 MPa in control conditions, but the compliance of the capillaries increased in 5/6‐nephrectomized conditions due to a 23.3% increase in the baseline glomerular capillary diameters. We found that glomerular capillary wall strain was increased approximately threefold in 5/6‐nephrectomized conditions over control, which may deleteriously affect both mesangial cells and podocytes. The magnitudes of strain in model simulations of 5/6‐nephrectomized conditions were consistent with magnitudes of strain that elicit podocyte hypertrophy and actin cytoskeleton reorganization in vitro.

**Conclusions:**

Our findings indicate that glomerular capillary wall strain may deleteriously affect podocytes directly, as well as act in concert with other mechanical changes and environmental factors inherent to the in vivo setting to potentiate glomerular injury in severe renoprival conditions.

## 1 INTRODUCTION

The renal glomerulus is a compliant structure that changes in volume depending on the pressure at which the blood enters the filtering capillaries. Multiple experimental strategies have been employed to estimate the compliance of the glomerulus in response to changes in perfusion pressure and investigate how this compliance is affected by disease.^1^, ^2^, ^3^, ^4^ While these studies provided a wealth of information in terms of characterizing the bulk response of the glomerular tuft to changes in perfusion pressure, the elasticity of the walls of individual glomerular capillaries remains to be rigorously quantified. Furthermore, it is currently unknown how temporal changes in glomerular pressure translate to the magnitude of strain (stretch) of the individual glomerular capillary walls, and how these strains are distributed throughout the glomerular capillary network.

The magnitude of glomerular capillary wall strain is hypothesized to be of importance in numerous diseases in which glomerular capillary pressure is increased, such as diabetes,^5^ hypertension ^6^ and in conditions involving the loss of functioning nephrons.^7^ In vitro, cyclic mechanical strain deleteriously affects both podocytes ^8^, ^9^, ^10^, ^11^, ^12^, ^13^, ^14^ and mesangial cells,^12^, ^15^, ^16^ and stretching acts synergistically with additional factors such as hyperglycemia^17^ and TGF‐β1^8^ to compound these insults. With limited understanding of the magnitude of the strains on individual glomerular capillaries in vivo, the degree to which the glomerular cells are subjected to injurious levels of mechanical strain in pathophysiological conditions remains unclear. This is because the results of in vitro studies of podocyte strain are magnitude‐dependent, thus translating the results of these studies to the in vivo setting requires accurate estimation of the magnitudes of mechanical strain of the glomerular capillary walls in vivo.

Estimating the magnitudes of mechanical strain throughout the glomerular capillary network necessitates quantification of the compliance or elasticity of the glomerular capillary walls such that changes in glomerular pressure may be translated to changes in individual glomerular capillary diameters. Rat glomerular compliance, in terms of the degree to which the glomerulus increases (decreases) in total volume as a result of increases (decreases) in perfusion pressure, has been estimated previously.^1^, ^2^ Past studies of glomerular compliance involved the removal of the renal corpuscle from the tissue sample, immersion of the corpuscle in an osmotic solution after removing Bowman's capsule, and perfusion of the same solution at varying pressures to measure changes in glomerular volume.^1^ Experimentally, it is difficult to translate the results of these in vitro studies to in vivo conditions, in which Bowman's space pressure and a substantial colloid osmotic gradient hinder filtration across the capillary wall and the blood is, under physiological conditions, more viscous than the filtrate due to plasma protein concentration and hematocrit. Furthermore, this compliance relates to the change in total glomerular volume as a function of perfusion pressure and does not necessarily describe the compliance of individual glomerular capillaries.

Using a novel mathematical model of fluid flow and filtration in a compliant, anatomically accurate rat glomerular capillary network,^18^ we estimated the elasticity and the strain of the individual glomerular capillaries in 5/6‐nephrectomized (5/6‐Nx) conditions based on results from studies that quantified glomerular volumetric compliance as a function of perfusion pressure.^1^ We used our model to determine how these acute changes in the glomerular capillary diameters collectively affect glomerular volume, filtration and mechanical stress within the glomerular capillaries. We then compared our model predictions to the results of in vitro studies of podocyte strain to make inferences on the effect of 5/6‐Nx‐induced glomerular hypertension on podocyte structure and function.

## 2 MATHEMATICAL MODEL

We used a mathematical model of glomerular blood flow and filtration^18^ to estimate filtration dynamics and mechanical stress in the glomerular capillaries. This model was composed of an anatomically accurate rat glomerular capillary network, represented as a graph consisting of 320 glomerular capillary segments (graph edges) and 195 nodes at which capillaries branched and/or coalesced (graph vertices). The capillaries had a mean diameter of 8.3 μm and mean length of 20.9 μm. Maximum capillary length and diameter were 109 μm and 21.3 μm, respectively, and minimum capillary length and diameter were 2.5 and 2 μm, respectively. At baseline pressure, the total glomerular surface area and volume were 1.76 × 10^5^ μm^2^ and 5.14 × 10^5^ μm^3^, respectively.

On the length of each capillary segment, we used a model of glomerular filtration to calculate the capillary segment pressure profile and capillary segment glomerular filtration rate (CSGFR). A system of linear equations was used to calculate network node pressures which were then used as boundary conditions for the capillary model. Hematocrit was asymmetrically distributed throughout the network^19^ and was used to calculate the apparent blood viscosity in each capillary segment.^20^ The blood viscosity and filtration resistance were iteratively updated until the model reached convergence. Building on our previous work, we iteratively updated glomerular capillary diameters and lengths using stress‐strain relationships. We briefly describe each of these model components below.

### 2.1 Filtering capillary model

We modeled a glomerular capillary spanning from node *i* to node *j* as a cylindrical, permeable vessel of diameter *D_ij_* and length *L_ij_*. Variables tracked on the length of the capillary included *p_ij_* (*x*), the axial pressure profile; *Q*_ij_(*x*), the blood flow; *C_ij_* (*x*), the plasma protein concentration; Π*_ij_* (*x*), the colloid osmotic pressure; and *E_ij_*, the erythrocyte volume assumed constant on the length of the capillary. CSGFR*_ij_*, the capillary's filtration rate, was defined as:

(1) $${\mathrm{CSGFR}}_{a}=\frac{\int_{‐\frac{L_{\mathrm{ij}}}{2}}^{\frac{L_{\mathrm{ij}}}{2}}\left(p_{\mathrm{ij}}\left(x\right)‐p_{\mathrm{BS}}\right)dx}{R_{\mathrm{ij}}^{f}L_{\mathrm{ij}}},$$

where $R_{\mathrm{ij}}^{f}$ is the resistance of the glomerular capillary wall to filtration, and *p*_BS_ is Bowman's space pressure, which is assumed the same for all capillaries. To calculate the pressure profile on the length of the capillary, we solved the second‐order linear differential equation which we derived in a previous work to model the change of pressure on the length of a permeable capillary ^18^:

(2) $$\frac{d^{2}p_{\mathrm{ij}}}{dx^{2}}\left(x\right)‐a_{\mathrm{ij}}^{2}p_{\mathrm{ij}}\left(x\right)=‐a_{\mathrm{ij}}^{2}p_{\mathrm{BS}}.$$

The boundary conditions of equation 2 are *p_ij_* (‐*L_ij_*/2) = *p_i_* and *p_ij_* (*L*_ij_/2) = *p_j_*, for *p_i_* and *p_j_* the pressure at nodes *i* and *j*, respectively; $a_{\mathrm{ij}}^{2}=R_{\mathrm{ij}}/R_{\mathrm{ij}}^{f}L_{\mathrm{ij}}^{2}$, for *R_ij_* the resistance of the capillary. This equation was derived assuming that the filtration resistance $R_{\mathrm{ij}}^{f}$ is constant along the length of the capillary, such that the capillary may be sectioned into M sections of length *L_ij_*/*M*, each filtering with a filtration resistance of $M_{\mathrm{ij}}^{f}$. By ensuring flux balance between each of these segments while taking into account fluid loss due to filtration at each segment, and taking *M* → ∞ we obtained equation 2, as described in our previous work.^18^ Solving equation 2, we obtained the pressure profile on the length of the capillary:

(3) $$p_{\mathrm{ij}}\left(x\right)=p_{\mathrm{BS}\;}+\left(\frac{p_{i}+p_{j}}{2}‐p_{\mathrm{BS}\;}\right)\frac{\cosh\left(a_{\mathrm{ij}}x\right)}{\cosh\left(\frac{a_{\mathrm{ij}}L_{\mathrm{ij}}}{2}\right)}+\left(\frac{p_{j}‐p_{i}}{2}\right)\frac{\sinh\left(a_{\mathrm{ij}}x\right)}{\sinh\left(\frac{a_{\mathrm{ij}}L_{\mathrm{ij}}}{2}\right)}.$$

The relationship between *p_ij_* (*x*) and *Q_ij_* (*x*) was as follows:

(4) $$Q_{\mathrm{ij}}\left(x\right)=‐\frac{L_{\mathrm{ij}}\;dp_{\mathrm{ij}}}{R_{ij\;}dx}\left(x\right).$$

Thus,

(5) $$Q_{\mathrm{ij}}\left(x\right)=‐\frac{L_{\mathrm{ij}}}{R_{\mathrm{ij}}}\left(\frac{p_{i}+p_{j}}{2}‐p_{\mathrm{BS}}\right)\frac{a_{\mathrm{ij}}\sinh\left(a_{\mathrm{ij}}x\right)}{\cosh\left(\frac{a_{\mathrm{ij}}L_{\mathrm{ij}}}{2}\right)}‐\frac{L_{\mathrm{ij}}}{R_{\mathrm{ij}}}\left(\frac{p_{j}‐p_{i}}{2}\right)\frac{a_{\mathrm{ij}}\cosh\left(a_{\mathrm{ij}}x\right)}{\sinh\left(\frac{a_{\mathrm{ij}}L_{\mathrm{ij}}}{2}\right)}.$$

We modeled the filtration at each point on the length of the capillary as:

(6) $$\frac{dQ_{\mathrm{ij}}}{\mathrm{dx}}\left(x\right)=‐k\pi D_{\mathrm{ij}}\left(p_{\mathrm{ij}}\left(x\right)‐p_{\mathrm{BS}}‐\Pi_{\mathrm{ij}}\left(x\right)\right),$$

for *k* the hydraulic conductivity of the capillary wall, defined as the permeability of the glomerular filtration barrier to water, and Π*_ij_* (*x*) the colloid osmotic pressure as a function of plasma protein concentration *C_ij_* (*x*):

(7) $$\Pi_{\mathrm{ij}}\left(x\right)=2.1C_{\mathrm{ij}}\left(x\right)+0.16C_{\mathrm{ij}}^{2}\left(x\right)+0.009C_{\mathrm{ij}}^{3}\left(x\right).$$

Assuming mass balance,

(8) $$C_{\mathrm{ij}}\left(x\right)=C_{\mathrm{ij}}\left(‐\frac{L_{\mathrm{ij}}}{2}\right)\frac{Q_{\mathrm{ij}}\left(‐\frac{L_{\mathrm{ij}}}{2}\right)‐E_{\mathrm{ij}}}{Q_{\mathrm{ij}}\left(x\right)‐E_{\mathrm{ij}}}.$$

Plasma proteins were assumed to not escape the glomerular capillary lumen ^21^ thus the colloid osmotic pressure only hinders filtration. To ensure consistency of the model equations 1 and 6, we required that:

(9) $$\frac{\int_{‐\frac{L_{\mathrm{ij}}}{2}}^{\frac{L_{\mathrm{ij}}}{2}}\left(p_{\mathrm{ij}}\left(x\right)‐p_{\mathrm{BS}}\right)dx}{R_{\mathrm{ij}}^{f}L_{\mathrm{ij}}}=\underset{‐\frac{L_{\mathrm{ij}}}{2}}{\int^{\frac{L_{\mathrm{ij}}}{2}}}k\pi D_{\mathrm{ij}}\left(p_{\mathrm{ij}}\left(x\right)‐p_{\mathrm{BS}}‐\Pi_{\mathrm{ij}}\left(x\right)\right)dx.$$

To maintain this equality, we note that $R_{\mathrm{ij}}^{f}$ was not fixed but could be iteratively updated so that this equality held, as shown in the model algorithm below. The permeability of the glomerular capillaries was determined by the hydraulic conductivity *k*, which was fixed and was assumed to be the same for all capillaries in the network (Table 1).

**TABLE 1:** Parameters used in the simulations of mechanical forces and filtration in the glomerulus in control and 5/6‐Nx with associated references

Condition	R_a_ (10^10^ dyn s cm^‐5^)	R_e_ (10^10^ dyn s cm^‐5^)	C_a_ (g/dl)	p_BS_ (mmHg)
Control	7.2	1.8	5.2	13
5/6‐Nx	2.9	0.8	5.3	12
Ref	^18^	^18^	^7^	^7^

DBP (mmHg)	SBP (mmHg)	t^bm^ (nm)	k (10^−5^ nl min^−1^ mmHg^−1^ μm^−2^)	ϕ_V_ (% mmHg^‐1^)	Increase in baseline diameter (%)
113	136	149.82	1.6	0.175	0
111	165	146.99	2.4	0.22	23.3
7, 26, 27		^40^	^18^	^1^	^25^

Abbreviations: *C*_a_, afferent plasma protein concentration; DBP, diastolic blood pressure in each case; *k*, glomerular capillary wall hydraulic conductivity; *p*_BS_, Bowman's Space pressure; *R*_a_, afferent resistance; *R*_e_, efferent resistance; SBP, systolic blood pressure in each case; *t* ^bm^, glomerular basement membrane thickness; ϕ_V_, total relative glomerular volumetric compliance.

Additional parameters, including podocyte foot process height and width, are included in our previous work.^18^

### 2.2 Network model

We extrapolated equations (1), (2), (3), (4), (5), (6), (7), (8) to the entire network of capillaries by imposing boundary conditions at the inlet of the afferent arteriole and outlet of the efferent arteriole, denoted by subscripts “a” and “e,” respectively. Pressures were calculated at network nodes with boundary conditions *p_a_* and *p_e_*, set equal to blood pressure and peritubular capillary pressure, respectively. Afferent and efferent arterioles were assumed to have fixed resistances *R_a_* and *R_e_* that do not change, as the model is steady state. To calculate pressures at node *i*, we assumed conservation of blood flow at each network node. Thus for *J* the set of nodes *j* connected to node i, we solved the conservation of flow equation:

(10) $$\sum_{j\in J}Q_{\mathrm{ij}}\left(‐\frac{L_{\mathrm{ij}}}{2}\right)=0,$$

For *Q_ij_* as described in equation 5. We evaluated *Q_ij_* at *x* = −*L_ij_*/2 in this equation because this position corresponded to the location at which blood is flowing into or out of node *i* as opposed to node *j*, at which *x* = +*L_ij_*/2. Equation 10 describes a linear system that we used to solve for all node pressures p_i_ given pressure boundary conditions *p_a_* and *p_e_*. Using the node pressures as boundary conditions for each corresponding capillary, we obtained the filtration rate, pressure and flow profiles of each capillary using equations 1, 3 and 5, respectively.

We obtained the plasma protein concentration profiles on each capillary segment using the boundary condition *C_a_* to denote the plasma protein concentration at the inlet of the afferent arteriole, set equal to systemic plasma protein concentration (Table 1). We assumed conservation of plasma protein mass and perfect mixing at each network node, thus if we let *K* be the set of nodes *k* upstream of and connected to node *i*, and *J* be the set of nodes *j* downstream of and connected to node *i*, then for all nodes *j* in *J* we defined:

(11) $$C_{\mathrm{ij}}\left(‐\frac{L_{\mathrm{ij}}}{2}\right)=\frac{\sum_{k\epsilon K}C_{\mathrm{ik}}\left(\frac{L_{\mathrm{ik}}}{2}\right)\left(Q_{\mathrm{ik}}\left(\frac{L_{\mathrm{ik}}}{2}\right)‐E_{\mathrm{ik}}\right)}{\sum_{k\epsilon K}\left(Q_{\mathrm{ik}}\left(\frac{L_{\mathrm{ik}}}{2}\right)‐E_{\mathrm{ik}}\right)}.$$

Erythrocyte flow *E_ij_* was determined for each capillary segment using the boundary condition *E_a_* which denotes the flow of erythrocyte volume at the inlet of the afferent arteriole, calculated using systemic hematocrit.^22^ A nonlinear function was used to distribute the erythrocyte flow at the network nodes.^19^ This function was dependent upon daughter branch diameters and hematocrit in the feeding vessel. Capillary hematocrit (*H_t_*)*_ij_* was defined as:

(12) $${{(H}_{t})}_{\mathrm{ij}}=\frac{E_{\mathrm{ij}}}{{\bar{Q}}_{\mathrm{ij}}\left(x\right)},$$

Where the bar operator (–) indicates averaging *Q_ij_* (*x*) on the length of the vessel. Thus, given pressure boundary conditions *p_a_* and *p_e_*, plasma protein boundary condition *C_a_* and erythrocyte boundary condition *E_a_*, we calculated flow and filtration in each capillary segment in the glomerular capillary network.

### 2.3 Apparent viscosity and mechanical stress equations

To determine the capillary resistance *R_ij_* used in the equations above, we took into account the non‐Newtonian characteristics of blood, namely, its mutable viscosity. Assuming Poiseuille flow:

(13) $$R_{\mathrm{ij}}=\frac{128\mu_{\mathrm{ij}}L_{\mathrm{ij}}}{\pi D_{\mathrm{ij}}^{4}},$$

where *μ_ij_* was the apparent viscosity of the blood as a function of hematocrit:

(14) $$\mu_{\mathrm{ij}}=\mu_{\mathrm{ij}}^{\mathrm{pl}}\lambda \left(D_{\mathrm{ij}},{{(H}_{t})}_{\mathrm{ij}}\right).$$

In this formulation, $\mu_{\mathrm{ij}}^{\mathrm{pl}}$ had a linear relationship with the average plasma protein concentration on the length of the vessel ^23^ and *λ* was an empirically derived nonlinear function of the vessel diameter and hematocrit.^20^ In addition to $R_{\mathrm{ij}}^{f}$, *μ_ij_* was updated iteratively so that resistance of the capillaries was recalculated with each iteration. We estimated glomerular capillary shear stress, τ, by taking into account loss of flow due to filtration on the length of the vessel:

(15) $$\tau_{\mathrm{ij}}=\frac{32\mu_{\mathrm{ij}}{\bar{Q}}_{\mathrm{ij}}\left(x\right)}{\pi D_{\mathrm{ij}}^{3}}.$$

Circumferential stress, also known as hoop stress, denoted by *σ*, was calculated using the Young‐Laplace equation:

(16) $$\sigma_{\mathrm{ij}}=\frac{D_{\mathrm{ij}}\left({\bar{p}}_{\mathrm{ij}}‐p_{\mathrm{BS}}\right)}{2t_{\mathrm{ij}}},$$

where the bar operator again indicates averaging along the length of the capillary and *t_ij_* was the capillary wall thickness, taken to be a function of the endothelial cell layer thickness *t^e^*, the glomerular basement membrane thickness *t^bm^*, the minimum podocyte layer thickness $t_{\min}^{\mathrm{pod}}$, and the podocyte foot process width, *w* ^pod^ and height, *h* ^pod^:

(17) $$t_{\mathrm{ij}}=t^{e}+t^{\mathrm{bm}}+t_{\text{min}}^{\text{pod}}+\frac{h^{\text{pod}}}{2}+\frac{1}{\pi D_{\mathrm{ij}}}\int_{0}^{\pi D_{\mathrm{ij}}}\frac{h^{\text{pod}}}{2}\cos\left(\frac{2\pi }{w^{\text{pod}}}\left(x‐\frac{w^{\text{pod}}}{2}\right)\right)dx.$$

The basement membrane thickness, *t^bm^* changed slightly in 5/6‐Nx (Table 1). The remaining thicknesses and dimensions of the podocyte foot processes were the same as in our previous work.^18^ A change in the circumferential and/or longitudinal stress from the baseline value was responsible for strain of the glomerular capillaries, as described below.

### 2.4 Glomerular capillary compliance

To calculate strain of the glomerular capillary wall, we developed a constitutive relation assuming that the glomerular filtration barrier was an incompressible, neo‐Hookean solid with the following strain energy density function ^24^:

(18) $$W=\frac{E}{3}\left(\varepsilon_{r}+\varepsilon_{\theta }+\varepsilon_{x}\right),$$

for E the Young's modulus of the glomerular capillary wall, and *ε_r_*, *ε_θ_*, and *ε_x_* the radial, circumferential and longitudinal strains, respectively. A neo‐Hookean solid model was used based on the assumption that the nonlinear terms characteristic of soft tissue biomechanics were unnecessary when strains were assumed to be lower than 10%.^24^ Nonzero *ε_r_*, *ε*_θ_, and *ε_x_* corresponded to pressure‐induced changes in *t_ij_*, *D_ij_*, and *L_ij_*, respectively. From our strain energy density function, we defined the stresses *σ_r_*, *σ*_θ_, and *σ_x_* such that

(19) $$\sigma_{r}=\frac{E}{3}\varepsilon_{r},$$

(20) $$\sigma_{\theta }=\frac{E}{3}\varepsilon_{\theta },$$

(21) $$\sigma_{x}=\frac{E}{3}\varepsilon_{x}.$$

Using these relations, we estimated changes in *t_ij_*, *D_ij_*, and *L_ij_* due to the stresses imposed on the glomerular capillary walls by changes in pressure. To notate these changes, the superscript “0” is used to indicate the variable when the blood pressure is equal to MAP. Assuming incompressibility of the capillary wall, we determined the radial strain as a function of the circumferential and longitudinal strains:

(22) $$\varepsilon_{r}=\frac{1}{\left(\varepsilon_{\theta }+1\right)\left(\varepsilon_{x}+1\right)}‐1.$$

The circumferential and longitudinal stresses, *σ_θ_* and *σ_x_*, were estimated using equations of stress in a thin‐walled cylinder. We assume a thin wall of the capillaries because the glomerular capillary diameters, measured in μm, are at least an order of magnitude larger than the wall thickness, measured in nm. For a given capillary segment *ij*, we let

(23) $$t_{\mathrm{ij}}=t_{\mathrm{ij}}^{0}\frac{D_{\mathrm{ij}}^{0}L_{\mathrm{ij}}^{0}}{D_{\mathrm{ij}}L_{\mathrm{ij}}},$$

(24) $$\frac{D_{\mathrm{ij}}\left({\bar{p}}_{\mathrm{ij}}‐p_{\mathrm{BS}}\right)}{2t_{\mathrm{ij}}}‐\frac{D_{\mathrm{ij}}^{0}\left({\bar{p}}_{\mathrm{ij}}^{0}‐p_{\mathrm{BS}}\right)}{2t_{\mathrm{ij}}^{0}}=\frac{E}{3}\left(\frac{D_{\mathrm{ij}}}{D_{\mathrm{ij}}^{0}}‐1\right),$$

(25) $$\frac{D_{\mathrm{ij}}\left({\bar{p}}_{\mathrm{ij}}‐p_{\mathrm{BS}}\right)}{4t_{\mathrm{ij}}}‐\frac{D_{\mathrm{ij}}^{0}\left({\bar{p}}_{\mathrm{ij}}^{0}‐p_{\mathrm{BS}}\right)}{4t_{\mathrm{ij}}^{0}}=\frac{E}{3}\left(\frac{L_{\mathrm{ij}}}{L_{\mathrm{ij}}^{0}}‐1\right).$$

Using these equations we solved for *t_ij_*, *D_ij_*, and *L_ij_* as functions of $t_{\mathrm{ij}}^{0}$, $D_{\mathrm{ij}}^{0}$, and $L_{\mathrm{ij}}^{0}$ taking into account the change of pressure from $p_{\mathrm{ij}}^{0}$ to *p_ij_*:

(26) $$t_{\mathrm{ij}}=t_{\mathrm{ij}}^{0}\frac{D_{\mathrm{ij}}^{0}L_{\mathrm{ij}}^{0}}{D_{\mathrm{ij}}L_{\mathrm{ij}}},$$

(27) $$L_{\mathrm{ij}}=L_{\mathrm{ij}}^{0}\left(\frac{3{\left(D_{\mathrm{ij}}^{0}\right)}^{2}\left({\bar{p}}_{\mathrm{ij}}^{0}‐p_{\mathrm{BS}}\right)‐4Et_{\mathrm{ij}}^{0}D_{\mathrm{ij}}^{0}}{3{\left(D_{\mathrm{ij}}\right)}^{2}\left({\bar{p}}_{\mathrm{ij}}‐p_{\mathrm{BS}}\right)‐4Et_{\mathrm{ij}}^{0}D_{\mathrm{ij}}^{0}}\right),$$

(28) $${{(D}_{\mathrm{ij}})}^{3}+{{D_{\mathrm{ij}}^{0}(D}_{\mathrm{ij}})}^{2}‐\frac{4Et_{\mathrm{ij}}^{0}D_{\mathrm{ij}}^{0}}{3\left({\bar{p}}_{\mathrm{ij}}‐p_{\mathrm{BS}}\right)}D_{\mathrm{ij}}‐\;\frac{2{{(D}_{\mathrm{ij}}^{0})}^{3}\left({\bar{p}}_{\mathrm{ij}}^{0}‐p_{\mathrm{BS}}\right)}{\left({\bar{p}}_{\mathrm{ij}}‐p_{\mathrm{BS}}\right)}+\;\frac{4Et_{\mathrm{ij}}^{0}{{(D}_{\mathrm{ij}}^{0})}^{2}}{3\left({\bar{p}}_{\mathrm{ij}}‐p_{\mathrm{BS}}\right)}=0.$$

Thus *t_ij_* and *L_ij_* were functions of *D_ij_*, which was determined by solving for the real root of the cubic polynomial in equation 28.

Since E of the glomerular capillary walls was unknown, we needed to estimate it by fitting the model to compliance data obtained by Cortes et al.^1^ This compliance, denoted ϕ_V_, was calculated based on the change of the glomerular volume, *V*_G_, over basal glomerular volume, *V*_0_, in response to a change in proximal intraglomerular pressure, PIP_G_ over basal proximal intraglomerular pressure, PIP_0_. Proximal intraglomerular pressure was the pressure measured at the entrance of the glomerulus in lieu of the glomerular capillary pressure in the experimental setup of Cortes et al. We defined

(29) $$\phi_{V}=\frac{\frac{V_{G}}{V_{0}}‐1}{{\mathrm{PIP}}_{G}‐{\mathrm{PIP}}_{0}},$$

which represents the percentage change in volume per mmHg increment in PIP. Cortes et al. used a PIP_0_ of 50 mmHg, and a PIP_G_ of 120 mmHg. Based on data from Cortes et al.,^1^ ϕ_V_ equaled 0.175% mm/Hg in control conditions and 0.220% mm/Hg in 5/6‐Nx conditions (Table 1). This compliance describes the change of the entirety of the glomerular capillary network volume to changes in PIP; however, our formulation for glomerular capillary compliance required that we use a specific Young's modulus, E to alter glomerular capillary dimensions. We took a simulation‐based approach of estimating the Young's modulus, whereby we tried different values of E in the model until the model's total volumetric compliance was the same as that measured by Cortes et al. in control and 5/6‐nephrectomized conditions. This process is detailed in our results.

### 2.5 Model algorithm

**FIGURE 1:**
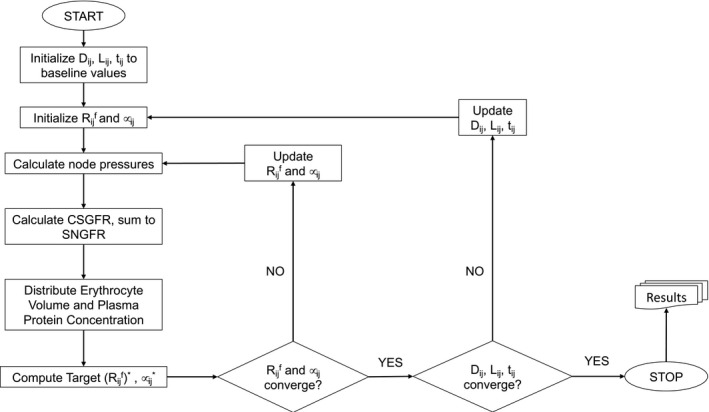
A flow chart schematic detailing the order of algorithm steps. The algorithm is composed of two loops: the inner loop updates capillary filtration resistances and viscosities, $R_{\mathrm{ij}}^{f}$ and *μ_ij_*, respectively, and the outer loop updates the capillary diameters *D_ij_*, length *L_ij_* and wall thickness *t*_ij_ based on the Young's modulus, E. The algorithm concludes when all five variables converge

The algorithm employed in our model formulation was composed of two nested loops, as shown in Figure 1. The inner loop involved the iterative updating of the filtration resistance $R_{\mathrm{ij}}^{f}$ and the apparent blood viscosity *μ_ij_* until these variables converged, at which point the algorithm proceeded to the second loop which involved updating glomerular capillary diameters *D_ij_*, lengths *L_ij_* and thicknesses *t_ij_* taking into account glomerular capillary compliance. We briefly describe the main equations of the algorithm, with more details of the algorithm steps provided in our previous work.^18^ The superscript “*n*” is used to indicate the iteration of the inner loop, and the superscript “m” is used to indicate the iteration of the outer loop. For the inner loop of the algorithm, equations 9 and 14 were used to calculate target values for $R_{\mathrm{ij}}^{f}$ and *μ_ij_*, respectively:

(30) $${{(R}_{\mathrm{ij}}^{f})}^{*}=\frac{\int_{\frac{{‐L}_{\mathrm{ij}}}{2}}^{\frac{L_{\mathrm{ij}}}{2}}\left(p_{\mathrm{ij}}^{n}\left(x\right)‐p_{\mathrm{BS}}\right)dx}{k\pi D_{\mathrm{ij}}^{m}L_{\mathrm{ij}}^{m}\int_{\frac{{‐L}_{\mathrm{ij}}}{2}}^{\frac{L_{\mathrm{ij}}}{2}}\left(p_{\mathrm{ij}}^{n}\left(x\right)‐p_{\mathrm{BS}}‐\Pi_{\mathrm{ij}}^{n}\left(x\right)\right)dx},$$

(31) $$\mu_{\mathrm{ij}}^{*}=\mu_{\mathrm{pl}}^{n}\lambda \left(D_{\mathrm{ij}}^{m},{\left(H_{t}\right)}_{\mathrm{ij}}^{n}\right).$$

These target values were then used to update $R_{\mathrm{ij}}^{f}$ and μ*_ij_* as follows:

(32) $$\mu_{\mathrm{ij}}^{n+1}=\mu_{\mathrm{ij}}^{n}+\frac{\mu_{\mathrm{ij}}^{*}‐\mu_{\mathrm{ij}}^{n}}{\alpha },$$

(33) $${{(R}_{\mathrm{ij}}^{f})}^{n+1}={{(R}_{\mathrm{ij}}^{f})}^{n}+\frac{{{(R}_{\mathrm{ij}}^{f})}^{*}‐{{(R}_{\mathrm{ij}}^{f})}^{n}}{\alpha },$$

For *α* a smoothing parameter to control the magnitude by which $R_{\mathrm{ij}}^{f}$ and *μ_ij_* may change so as to avoid iterative oscillations in filtration and/or viscosity throughout the network. Convergence of the inner loop was satisfied when:

(34) $$\max_{\mathrm{ij}}\frac{{|\mu }_{\mathrm{ij}}^{n+1}‐\mu_{\mathrm{ij}}^{n}|}{\mu_{\mathrm{ij}}^{n}}<\delta_{\mu },$$

(35) $$\max_{\mathrm{ij}}\frac{{{|(R}_{\mathrm{ij}}^{f})}^{n+1}‐{{(R}_{\mathrm{ij}}^{f})}^{n}|}{{{(R}_{\mathrm{ij}}^{f})}^{n}}<\delta_{R},$$

For *δ_μ_* = *δ_R_* = 1 × 10^−5^. In the outer loop of the algorithm, vessel diameters, lengths and wall thicknesses were updated according to equations (26), (27), (28):

(36) $$t_{\mathrm{ij}}^{m+1}=t_{\mathrm{ij}}^{0}\frac{D_{\mathrm{ij}}^{0}L_{\mathrm{ij}}^{0}}{D_{\mathrm{ij}}^{m+1}L_{\mathrm{ij}}^{m+1}},$$

(37) $$L_{\mathrm{ij}}^{m+1}=L_{\mathrm{ij}}^{0}\left(\frac{3{\left(D_{\mathrm{ij}}^{0}\right)}^{2}\left({\bar{p}}_{\mathrm{ij}}^{0}‐p_{\mathrm{BS}}\right)‐4Et_{\mathrm{ij}}^{0}D_{\mathrm{ij}}^{0}}{3{\left(D_{\mathrm{ij}}^{m+1}\right)}^{2}\left({\bar{p}}_{\mathrm{ij}}^{m}‐p_{\mathrm{BS}}\right)‐4Et_{\mathrm{ij}}^{0}D_{\mathrm{ij}}^{0}}\right),$$

(38) $${{(D}_{\mathrm{ij}}^{m+1})}^{3}+D_{\mathrm{ij}}^{0}{\left(D_{\mathrm{ij}}^{m+1}\right)}^{2}‐\frac{4Et_{\mathrm{ij}}^{0}D_{\mathrm{ij}}^{0}}{3\left({\bar{p}}_{\mathrm{ij}}^{m}‐p_{\mathrm{BS}}\right)}D_{\mathrm{ij}}^{m+1}‐\;\frac{2{{(D}_{\mathrm{ij}}^{0})}^{3}\left({\bar{p}}_{\mathrm{ij}}^{0}‐p_{\mathrm{BS}}\right)}{\left({\bar{p}}_{\mathrm{ij}}^{m}‐p_{\mathrm{BS}}\right)}+\;\frac{4Et_{\mathrm{ij}}^{0}{{(D}_{\mathrm{ij}}^{0})}^{2}}{3\left({\bar{p}}_{\mathrm{ij}}^{m}‐p_{\mathrm{BS}}\right)}=0.$$

In the algorithm steps equation 38 was solved to update *D_ij_* and the updated *D_ij_* was used to update *L_ij_* and *t_ij_* using equations 37 and 36, respectively. The outer loop of the algorithm converged when

(39) $$\max_{\mathrm{ij}}\frac{{|t}_{\mathrm{ij}}^{m+1}‐t_{\mathrm{ij}}^{m}|}{t_{\mathrm{ij}}^{m}}<\delta_{t},$$

(40) $$\max_{\mathrm{ij}}\frac{{|L}_{\mathrm{ij}}^{m+1}‐L_{\mathrm{ij}}^{m}|}{L_{\mathrm{ij}}^{m}}<\delta_{L},$$

(41) $$\max_{\mathrm{ij}}\frac{{|D}_{\mathrm{ij}}^{m+1}‐D_{\mathrm{ij}}^{m}|}{D_{\mathrm{ij}}^{m}}<\delta_{D},$$

For *δ_t_* = *δ_L_* = *δ_D_* = 1 × 10^−5^.

## 3 RESULTS

### 3.1 Estimating glomerular capillary wall E

The glomerular compliance data reported by Cortes et al.^1^ described the change in total glomerular capillary network volume as a function of PIP, and not the change in volume or diameter of individual capillaries. We took a simulation‐based approach to estimate the glomerular capillary wall Young's modulus, E by trying different values of E in the model under the experimental conditions employed by Cortes et al; in our model, we set Bowman's space pressure, efferent arteriole resistance and outlet pressure to 0, and assumed zero osmotic pressure gradient across the capillary walls. We perfused our model glomerulus with a hematocrit‐free fluid with a viscosity of 1 cP. We assumed that all glomerular capillaries had the same elasticity, and we varied E to minimize δ*_E_*, the error between our model‐calculated total glomerular volumetric compliance and glomerular volumetric compliance values obtained by Cortes et al:

(42) $$\delta_{E}=\left|\frac{V_{G}‐V_{0}}{V_{0}}‐\phi_{V}\left({\mathrm{PIP}}_{G}‐{\mathrm{PIP}}_{0}\right)\right|.$$

**FIGURE 2:**
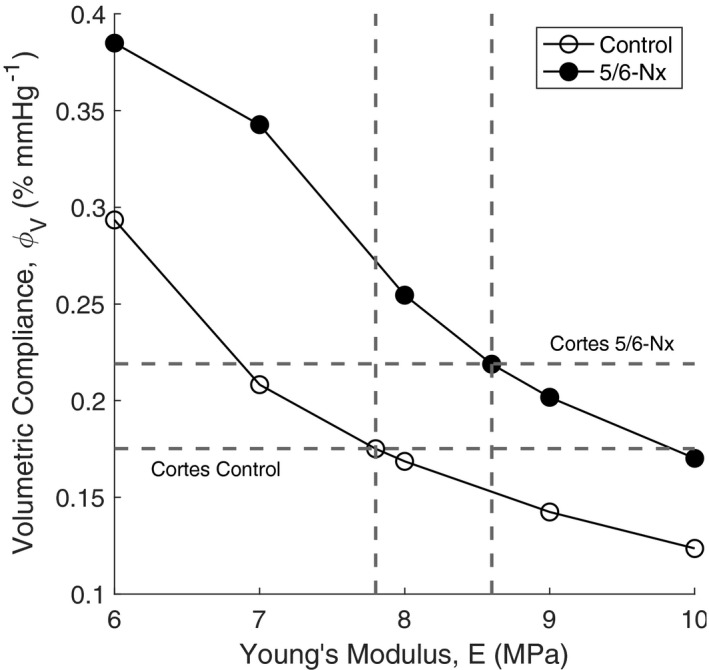
The relationship between the glomerular capillary wall Young's modulus, E and the total relative glomerular compliance, ϕ_V_, is determined by conducting simulations with different values of E. Both control simulations (open circles) and 5/6‐Nephrectomy simulations (5/6‐Nx, filled circles) are considered, and a substantial shift between the curves is evident. Horizontal dashed lines touch the vertical axis at the values of volumetric compliance measured experimentally by Cortes et al.^1^ in control and 5/6‐Nx. Vertical dashed lines touch the horizontal axis at values of Young's moduli that produce the same total glomerular volumetric compliance as that found by Cortes et al. Intersections of the dashed lines with their respective curves are marked with an additional simulation point

The model glomerular volume was calculated by summing the volumes of all glomerular capillaries. PIP was calculated by taking the pressure at the outlet of the afferent arteriole. Using this optimization scheme, we found that in control conditions E = 7.8 MPa. We performed a sensitivity analysis to determine the relationship between *E* and ϕ_V_, and determined how changing the glomerular capillary wall E affects the compliance of the glomerular volume in response to alterations in PIP (Figure 2). We considered both control conditions and 5/6‐Nx conditions in which hypertrophy increased glomerular capillary diameters by 23.3% at baseline pressure.^25^ This increase in baseline diameter shifts the curve in Figure 2 to the right, which is why the glomerular capillaries are more compliant even with a higher E in 5/6‐Nx over control (Table 2).

**TABLE 2:** Elastic modulus, glomerular hemodynamics and mechanical stresses in control and 5/6‐Nx conditions

Condition	E (MPa)	*P*_GC_ (mmHg)	*Q*_A_ (nl/min)	SNGFR (nl/min)	*V*_G_ (10^5^ μm^3^)	Hoop stress (kPa)	Shear stress (dyn/cm^2^)
Control	7.8	51.5 ± 4.1	111.7 ± 11.3	35.5 ± 7.3	4.02 ± 0.03	62.4 ± 7.0	33.7 ± 3.5
5/6‐Nx	8.6	57.0 ± 9.9	309.2 ± 67	97 ± 38	6.1 ± 0.14	90.9 ± 21.2	43.2 ± 9.4

Abbreviations: E, Young's modulus of the glomerular capillary wall; *P*_GC_, glomerular capillary pressure; *Q*_A_, afferent plasma flow; SNGFR, single nephron glomerular filtration rate; *V*_G_, total glomerular capillary volume.

Variables that change with changes in blood pressure are expressed as the mean value ±the range of values that that variable takes over the course of a change in blood pressure from DBP to SBP (SBP – DBP =23 mmHg for control and 55 mmHg for 5/6‐Nx ^26^, ^27^).

### 3.2 5/6‐nephrectomy simulations

**FIGURE 3:**
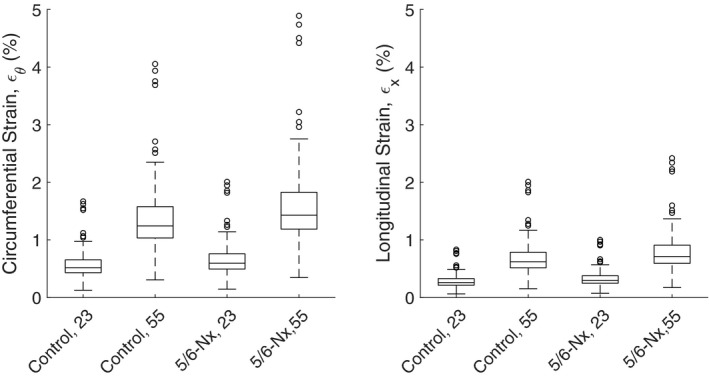
Capillary strain in control and 5/6‐Nx conditions. Labels on the *x*‐axis depict the condition in each simulation; “Control” and “5/6‐Nx” refer to the choice of E and change in baseline capillary diameters (Table 1), while the number (23 or 55) indicates the difference between the SBP and DBP in the simulation, in mmHg. The actual, physiological difference between SBP and DBP in control and 5/6‐Nx conditions is 23 and 55 mmHg, respectively, thus the first and fourth boxplots represent the model predictions in control and 5/6‐Nx conditions, respectively. Both circumferential and longitudinal strains were considered, while radial strain was found to be insignificant (below 1%) and thus was not included. The range of values depicted with boxplots and data points correspond to the strain values for each capillary in the glomerulus based on a single simulation, thus no statistical comparison is included

**FIGURE 4:**
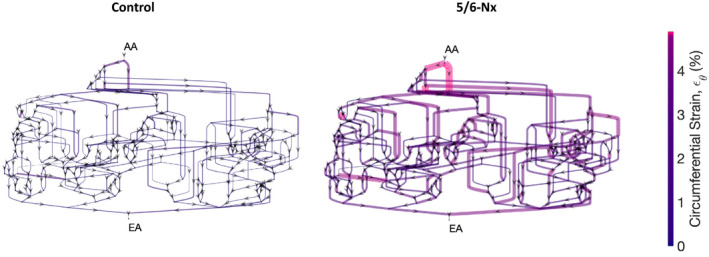
Distribution of glomerular capillary wall strains throughout the glomerular capillary network in control (left) and 5/6‐Nx conditions (right). Each graph segment represents a glomerular capillary, with arrows indicating flow direction in the model. “AA” and “EA” indicate afferent and efferent arterioles, respectively. The scale bar at right indicates the magnitude of glomerular capillary strain as a percentage change in diameter over the baseline diameter, and the thickness of each vessel is proportional to the strain on that vessel's wall, as well. The network strains are shown in control conditions to compare the strain magnitudes between the two conditions. Diagrams not drawn to scale

We quantified the magnitudes (Figure 3) and location (Figure 4) of glomerular capillary wall strain throughout the glomerular capillary network in response to an acute shift in blood pressure from diastolic blood pressure (DBP) to systolic blood pressure (SBP). Blood pressure was assumed to shift by SBP – DBP =23 mmHg and 55 mmHg in control and 5/6‐Nx conditions, respectively.^26^, ^27^ MAP for control and 5/6‐Nx conditions were 124 mmHg and 138 mmHg, respectively.^7^ Additional parameters are available in Table 1. Other than the alteration of the baseline diameters as discussed above, we did not change the network topology or the number of glomerular capillaries to simulate 5/6‐nephrectomized conditions, as to our knowledge there are no exhaustive tabulations of the glomerular capillary network structure in 5/6‐nephrectomized conditions.

In the simulations whose results are depicted in Figure 3, we considered control and 5/6‐Nx conditions (cases “Control, 23” and “5/6‐Nx, 55”) and examined the results in the case that the glomerulus undergoes structural changes associated with 5/6‐Nx (changing E and baseline diameters, as in Table 1) but the difference between SBP and DBP remains at control levels (23 mmHg). We also considered the opposite case in which the glomerulus has control levels of elasticity and capillary diameters but the difference between SBP and DBP is increased to 5/6‐Nx levels (55 mmHg). These cases are indicated by “5/6‐Nx, 23” and “Control, 55,” respectively. According to Figure 3, glomerular capillary wall strains (both circumferential and longitudinal) were greatly increased in 5/6‐Nx, particularly in the vessels closest to the afferent arteriole (Figure 4), and this was primarily the result of a larger difference between SBP and DBP in the 5/6‐Nx case. This is because when SBP‐DBP =23 mmHg, the increased compliance of the glomerulus associated with 5/6‐Nx conditions only meagerly increases strain over control conditions (Figure 3). In general, longitudinal strain was roughly equal to half of the circumferential strain, thus we only show a comparison of the two groups based on their circumferential strain in Figure 4, having confirmed that the analogous figure with longitudinal strain is almost identical to Figure 4.

We compared control and 5/6‐Nx groups with and without compliance to investigate the effect of glomerular capillary compliance on the mechanical stresses and hemodynamics in our simulations (Table 2). Error bars indicate the ranges of values that the variables take as blood pressure shifts from DBP to SBP in control and 5/6‐Nx conditions. Compliance had a negligible effect on the mean and range of glomerular hemodynamic indices and mechanical stresses, thus results of noncompliant simulations were not included in Table 2.

**FIGURE 5:**
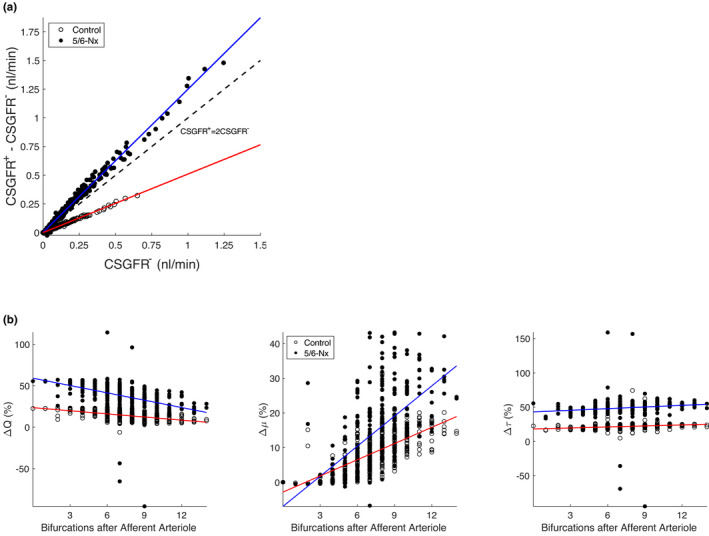
Localized changes in glomerular filtration and mechanics in control and 5/6‐Nx. A, Changes in CSGFR in each capillary segment over the course of a change in blood pressure from DBP to SBP (SBP – DBP =23 mmHg for control and 55 mmHg for 5/6‐Nx). Each data point corresponds to an individual capillary segment. Subscripts “+” and “−” indicate the value of CSGFR for blood pressure equal to SBP and DBP, respectively (Table 1). Red and blue lines fit the data for control and 5/6‐Nx conditions, respectively. The dashed line indicates the point at which the increase in blood pressure doubles the CSGFR. B, Relative changes in capillary blood flow *Q*, apparent viscosity *μ* and shear stress *τ* in control and 5/6‐Nx conditions, based on the location of the glomerular capillary in length along the glomerular network. Glomerular network length is quantified by counting the number of bifurcations on the shortest path from a capillary segment to the afferent arteriole. White and black points represent values pertaining to each capillary in the network in control and 5/6‐Nx conditions, respectively. Red and blue lines fit the data of control and 5/6‐Nx conditions, respectively

To further probe the effects of pressure fluctuations on the localized function and mechanics of the glomerulus in control and 5/6‐Nx conditions, we examined the changes in CSGFR (Figure 5A) and shear stress (Figure 5B). We found that in response to an increase in blood pressure, the capillaries that have the highest minimum CSGFR (denoted CSGFR^−^) will experience a proportionally larger change in CSGFR. Since the blood pressure shift in 5/6‐Nx was higher than that in control conditions, the acute changes in CSGFR in 5/6‐Nx were larger. We investigated blood pressure shift‐induced changes in shear stress throughout the glomerular capillary network as a function of length along the glomerulus (Figure 5B). The relative change in blood flow, ΔQ, was highest closest to the afferent arteriole, whereas Δμ was highest closer to the efferent arteriole, both of which were results of increased filtration and thus concentration of plasma proteins and hematocrit. As expected, the combination of these opposite gradients resulted in a roughly uniform change in shear stress, τ along the length of the glomerulus. The change in shear stress in 5/6‐Nx was larger due to the higher shift in blood pressure (Table 1) despite the increased diameters in the 5/6‐Nx case.

### 3.3 Sensitivity analysis

**FIGURE 6:**
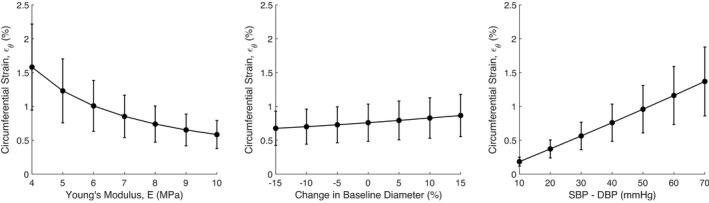
Sensitivity analysis of capillary wall circumferential strain, *ε_θ_* as a function of the capillary wall Young's modulus, E (left), the change in baseline capillary diameter (center) and the magnitude of the shift in blood pressure, equal to SBP – DBP (right). Error bars indicate the standard deviation of the strains experienced by the capillaries throughout the network

We conducted a sensitivity analysis to determine how glomerular capillary wall strain was influenced by parameters such as the wall Young's modulus, the baseline glomerular capillary diameters, and the magnitude of the shift in blood pressure (Figure 6). The reduction of E increased both the mean and standard deviation of strains throughout the glomerular capillary network. Increasing the baseline diameters increased the mean strain but did not affect the standard deviation, whereas alterations of the magnitude of the shift in blood pressure from DBP to SBP greatly influenced both the mean and standard deviation of strains throughout the network. Parameters governing the baseline thickness of the glomerular capillary wall, $t_{\mathrm{ij}}^{0}$, negligibly affected the magnitude of strain. This is reasonable because based on equations 27 and 28, $t_{\mathrm{ij}}^{0}$ should not appreciatively affect the changes in glomerular capillary length or diameter. In reality, the thickness of the wall would affect E and thus play a role in shaping the elasticity, however, because the wall thickness and E are uncoupled in our model, $t_{\mathrm{ij}}^{0}$ does not influence glomerular capillary wall elasticity on its own.

## 4 DISCUSSION

We developed a mathematical model of blood flow and filtration in a compliant glomerular capillary network to rigorously quantify the magnitudes of mechanical strain of the glomerular capillaries with and without loss of significant renal mass. Our model is novel in that it accounts for changes in glomerular capillary diameters in the distribution of erythrocytes and plasma throughout the capillary network, whereas previous anatomically accurate glomerular models have assumed fixed capillary diameters.^23^, ^28^ After parameterizing the model with data that related changes in perfusion pressure to glomerular volume,^1^ we estimated the magnitude of the strain of the glomerular capillary walls in a remnant glomerulus of a 5/6‐nephrectomized rat in response to physiological changes in blood pressure. Our simulations revealed that the median circumferential and longitudinal glomerular capillary wall strains were increased roughly threefold due to the increased glomerular capillary pressure and elasticity in 5/6‐Nx,^7^ and that these strains were highest in the vessels that branch off the afferent arteriole. The values of wall strain predicted by our model, particularly those pertaining to the vessels that branch off the afferent arteriole, are of a sufficient magnitude (approximately 4.9%) to affect podocyte structure and function in vitro.^14^

Mechanical strain deleteriously affects podocytes through numerous mechanisms.^9^, ^10^ When subjected to 5% biaxial strain in vitro, podocytes hypertrophy ^14^ and reorganize their actin cytoskeleton to accommodate the enhanced mechanical load.^29^, ^30^ However, increased levels of strain such as 10% biaxial strain are necessary to cause podocyte detachment from the glomerular basement membrane in vitro.^8^ Given this discrepancy, our simulations indicate that increases in strain may directly influence podocyte structure and function, and may also act in concert with other mechanical and biochemical changes in the glomerular environment to deleteriously affect podocyte stability.

Firstly, additional factors inherent to the in vivo setting, such as immune cell involvement, basement membrane stiffening and mesangial cell signaling, may play a role in the translation of glomerular capillary wall strain to podocyte apoptosis and foot process effacement. Since these factors were not present in the in vitro studies used to evaluate the podocyte reaction to strain, it is possible that 5% wall strain itself is sufficient to cause podocyte foot process effacement in vivo, when this magnitude of strain has not been found to be as deleterious to podocytes in vitro. Secondly, TGF‐β1 has a similarly if not more deleterious effect on podocytes than even 20% biaxial strain in vitro, and cyclic strain increases podocyte expression of the TGF‐β1 receptor in vitro.^8^ Due to a pronounced increase in afferent blood flow, glomerular capillary wall shear stress increases substantially in 5/6‐Nx (Table 2). In response to increased magnitudes of shear stress, endothelial cells increase secretion of TGF‐β1.^31^ As such, the *combination* of mechanical strain on podocytes and shear stress on endothelial cells may contribute to podocyte injury in 5/6‐Nx.

Finally, our results indicate that in 5/6‐Nx, the average CSGFR more than doubles with each shift increase in blood pressure, and that the change in an individual capillary's CSGFR is proportional to its minimum CSGFR (Figure 5A). The CSGFR is assumed to be proportional to the shear stress on the podocyte foot processes. Shear stress on the podocyte foot processes has been implicated in hyperfiltration‐mediated glomerular injury by destabilizing the podocyte actin cytoskeleton through the binding of prostaglandin E2 (PGE_2_) with the EP2 receptor.^32^ At 5% biaxial strain, podocytes increase production of COX‐2 and secretion of PGE_2_,^33^ which would theoretically enhance the destabilizing effect of shear stress on the podocyte actin cytoskeleton.

To simulate a compliant glomerulus that changes in volume as a function of perfusion pressure, we used data from a previous study that perfused isolated rat glomeruli *in vitro* to estimate their volumetric compliance.^1^ Thus, the Young's modulus used in our simulations was not obtained directly from measurements of the elasticity of individual capillaries. Instead, we assumed that all glomerular capillaries had the same elasticity, and we perfused our simulated glomerular model at different pressures to match the volumetric compliance data of Cortes et al. to a specific value of the Young's modulus of the glomerular capillary wall (Figure 2). We estimated the Young's modulus of the glomerular capillary walls to be 7.8 MPa in control conditions. This estimation is in stark contrast to previous measurements of glomerular elasticity, which estimate the glomerular Young's modulus to be 2–4 kPa.^3^, ^4^ This substantial discrepancy can be explained by the experimental methods used to estimate glomerular Young's moduli, including atomic force microscopy, capillary micromechanics and microindentation.^3^, ^4^ Firstly, all these experimental methods were employed to estimate the elastic modulus of the entire glomerular tuft, which includes mesangial tissue in addition to the capillaries themselves and thus may not be representative of an individual glomerular capillary. Secondly, these methods rely on deformation of the glomerular tissue to calculate the elastic modulus, which takes significantly less force than would be necessary to stretch the wall of a capillary by applying pressure from the luminal side.^10^ A Young's modulus of 7.8 MPa is higher than that of basement membranes found in mesenteric capillaries and venules undergoing small strain, at 5 MPa.^34^ This comparison corroborates our model predictions because these experimental measurements were for non‐glomerular capillary basement membranes with no cellular components, and the diameter of these capillaries was significantly higher than that of the majority of the capillaries in our model. In the glomerulus, the podocytes and mesangial cells are believed to significantly contribute to the stiffness of the glomerular tuft,^3^ thus a smaller diameter capillary with thicker walls due to these cellular components would theoretically have a higher Young's modulus.

In this study, estimating the Young's modulus of the glomerular capillary walls required us to calculate the volumetric compliance of an entire glomerulus based on previous data.^1^ To our knowledge, this data represents the only direct measurement of glomerular compliance as a function of perfusion pressure, thus it was necessary to use the total volume of our glomerular model to map the volumetric compliance data obtained by Cortes et al. to a value of the glomerular capillary wall Young's modulus. We used the volume of our model glomerulus, equal to the sum of the volumes of all the capillaries in the network, to map values of volumetric compliance to a Young's modulus of the capillary walls (Figure 2). We calculated our glomerulus model volume in this manner because we model the glomerulus as a network without considering the additional volume taken up by the space between capillaries. Assuming the additional volume between the capillaries increased proportionally to the capillary volume, this was not expected to greatly affect our estimation of the Young's modulus of the capillary walls because the volumetric compliance derived from the data obtained by Cortes et al. is made relative to the glomerular capillary volume at baseline PIP.

Inherent to our strategy of estimating the Young's modulus of the glomerular capillary walls was the assumption that the Young's modulus is homogenous throughout the network of glomerular capillaries, as it is not clear how the elastic modulus differs between different capillaries in the same glomerulus. Differences in capillary structure and cell types, in particular the ratio of mesangial cells to podocytes covering the capillary exterior, may result in differential elastic properties throughout the glomerular capillary network. Furthermore, the contractile capabilities of podocytes and mesangial cells, which may greatly influence the elastic moduli of the glomerular capillary walls and even counteract the wall distention,^35^ were not incorporated into the model. This aspect of glomerular capillary contractility would similarly be affected by the ratio of mesangial cells and podocytes covering the outside of the glomerular capillary. As such, the strain values predicted by our model may be *overestimated* in regions of the glomerulus that have more expansive mesangial coverage that would restrict the ballooning of capillaries and may be *underestimated* in the capillaries with less mesangial coverage where changes in pressure may have a stronger influence on capillary diameter.^35^ This limitation demonstrates the need for further investigation of the heterogeneity of glomerular capillary structure throughout the network such that differences in elasticity between different capillaries may be incorporated into the model.

We have focused our analysis on the potential effect of model‐predicted glomerular capillary wall strain on podocyte structure and function; however, other glomerular cell types such as mesangial cells also respond to mechanical strain in vitro.^15^, ^16^, ^17^ While our model accounts for the geometry of the glomerular capillary network,^36^ the mesangium is not modeled explicitly and thus it is still unclear to what degree mesangial cells are stretched in vivo in physiological and pathophysiological conditions. Future modeling efforts will seek to integrate a mesangial component into the glomerular tuft. Additional potential areas of interest include more detailed mathematical modeling of the tension between stretch and filtration in the glomerular filtration barrier ^37^ and the effect of strain on collagen deposition by podocytes and mesangial cells.^38^

In conclusion, we developed a mathematical model of blood flow and filtration in a compliant rat glomerulus which is novel in that it improves on previous anatomically accurate glomerular models by accounting for changes in glomerular capillary diameter in distributing blood flow throughout the network. We subjected the model to the hemodynamic conditions characteristic of remnant glomeruli in the 5/6‐Nx rat model ^7^ to estimate the magnitudes and locations of these strains within the glomerular capillary network, and we found that glomerular capillary wall strain is increased approximately threefold in 5/6‐Nx, which, based on in vitro data, may have a deleterious effect on podocytes, both directly and indirectly.

Furthermore, our model results indicate that the strain was highest in the vessels closest to the afferent arteriole, matching the pattern of perihilar glomerulosclerosis that is characterized by scarring and fibrosis of the vascular pole of the glomerulus at which both afferent and efferent arterioles are located.^39^ This is the variant of glomerulosclerosis that is most common in diseases associated with an increased hemodynamic burden on the remaining glomeruli after significant loss of functional nephrons, as detected in human biopsies and animal models.^39^ As such, our mathematical model, by taking the complex anatomy of the glomerular capillary network into account, allows for the identification of regions of the network most susceptible to injury based on network architecture, and in this case, our model results correlate closely with patterns of glomerulosclerosis characteristic of the disease condition we simulated. These results highlight the utility of mathematical modeling in elucidating mechanisms of glomerular injury in different pathophysiological hemodynamic conditions.

## 5 PERSPECTIVE

Our analyses indicate that there are substantial increases in strain of the glomerular capillary walls due to the increased pressure and remodeling of the remaining glomeruli that is associated with the significant loss of functional kidney mass. Our results indicate that this increased strain may deleteriously affect podocytes directly and may also act in concert with other factors inherent to the in vivo setting after significant loss of renal mass to disrupt podocyte structural integrity, thereby perpetuating glomerular injury and sclerosis.

## CONFLICT OF INTEREST

All authors declare no competing interests.

## AUTHOR CONTRIBUTIONS

O.R. and R.C. developed the mathematical model formulation. O.R. conducted model experiments and data analysis. L.G.N. advised on parameter selection and physiological relevance of model results. O.R. wrote the manuscript. O.R, R.C, and L.G.N. edited the manuscript.

## Data Availability

The data and mathematical models used to support the findings of this study are available upon request to the corresponding author.
